# Hirschsprung's Associated Enterocolitis (HAEC) Personalized Treatment with Probiotics Based on Gene Sequencing Analysis of the Fecal Microbiome

**DOI:** 10.1155/2018/3292309

**Published:** 2018-10-14

**Authors:** Georg Singer, Karl Kashofer, Christoph Castellani, Holger Till

**Affiliations:** ^1^Department of Paediatric and Adolescent Surgery, Medical University of Graz, Graz, Austria; ^2^Institute of Pathology, Medical University of Graz, Graz, Austria

## Abstract

Approximately 40% of children with Hirschsprung's disease (HD) suffer from Hirschsprung's associated enterocolitis (HAEC) despite correct surgery. Disturbances of the intestinal microbiome may play a role. Treatment with probiotics based on individual analyses of the fecal microbiome has not been published for HD patients with recurrent HAEC yet. A boy with trisomy 21 received transanal pull-through at the age of 6 months for rectosigmoid HD. With four years, he suffered from recurrent episodes of HAEC. The fecal microbiome was measured during three healthy and three HAEC episodes by next-generation sequencing. The patient was started on daily probiotics for 3 months; the fecal microbiome was measured weekly. The fecal microbiome differed significantly between healthy and HAEC episodes. HAEC episodes were associated with significant decreases of Actinobacteria and significant increases of Bacteroidetes and Proteobacteria. Probiotic treatment led to a significant increase of alpha diversity and a significant increase of *Bifidobacterium* and *Streptococcus* as well as decreases of *Rikenellaceae*, *Pseudobutyrivibrio*, *Blautia*, and *Lachnospiraceae*. A longitudinal observation of the microbiome has never been performed following correction of Hirschsprung's disease. Probiotic treatment significantly changed the fecal microbiome; the alterations were not limited to strains contained in the administered probiotics.

## 1. Introduction

Hirschsprung's disease (HD) is represented by a congenital segmental absence of the enteral nervous system in both the myenteric and submucosal plexus with variable proximal expression due to a failure of migration of neural crest cells during embryonic development [1]. The resulting intestinal obstruction is usually treated by surgical removal of the aganglionic bowel and a pull-through of unaffected ganglionic bowel.

Despite correct endorectal pull-through for HD, up to 40% of the patients continue to suffer from Hirschsprung's associated enterocolitis (HAEC) defined as a clinical condition with diarrhea, abdominal discomfort, fever, and eventually subsequent septic shock [2]. Nevertheless, the exact pathogenesis of HAEC still remains unclear. Considering the complex interrelation between the epithelium, the immune system, and the microbiome of the intestine, disturbances of the intestinal microbial composition may predispose a patient to develop HAEC independent of correct surgical treatment.

Currently, next-generation sequencing is widely applied in gastrointestinal studies and facilitates the comprehensive description of the whole genome of the intestinal microbiota. With reference to Hirschsprung's disease, however, there are a limited number of reports describing disruptions of the intestinal microbiome in patients suffering from HAEC when compared to healthy Hirschsprung's disease patients [3–5]. Additionally, it has been shown that treatment with probiotics not only significantly diminishes the incidence but also decreases the severity of HAEC [6]. These two findings support the hypothesis that disruptions of the intestinal microbiome may be associated with the development of HAEC.

Nevertheless, the available evidence is based on interindividual comparisons of the intestinal microbiome including patients with or without enterocolitis, respectively. An intraindividual comparison of the intestinal microbiome during episodes with and without enterocolitis has not been performed yet. Neither has been studied whether or not treatment of Hirschsprung's disease patients with probiotics alters the intestinal microbiome.

Therefore, the aim of the present report was to describe the intestinal microbiome of a patient suffering from Hirschsprung's disease during episodes with and without enterocolitis and during treatment with probiotics applying 16S rRNA gene next-generation sequencing.

## 2. Case Presentation

A three-year-old boy with a rectosigmoidal Hirschsprung's disease and trisomy 21 received laparoscopically assisted Georgeson pull-through operation at the age of six months. Histology confirmed normal ganglion cells at the site of the anastomosis. The initial postoperative course was unremarkable; especially, the anastomosis healed with neither stricture nor dehiscence. Nevertheless, the boy suffered from recurrent episodes of HAEC with diarrhea, abdominal distention, pain, and alterations of the general condition classified as grade I according to the APSA criteria [7].

### 2.1. Healthy and HAEC Episodes

Stool samples were taken for microbial assessment during three healthy episodes and three HAEC episodes, sampled to PSP spin stool DNA sample kits (Stratec Molecular GmbH, Berlin, Germany), and stored at −21°C until measurement. The microbiome analysis was performed in duplicates as already published [8]. Statistical analysis was performed using the compare_categories.py and the group_significance.py scripts of QIIME 1.8. These scripts implement several statistical methods for the analysis of strength and statistical significance of sample groupings or OTUs via the vegan and ape R packages. Category significance was calculated using the Adonis and ANOSIM tests, while OTU significance was calculated using the Kruskal–Wallis test.

Alpha diversity between healthy and HAEC episodes was not significantly different (Chao 1 Index: mean healthy episode 967, SD 94; mean HAEC episode 1,009, SD 72; *p*=0.432).

**Figure 1:**
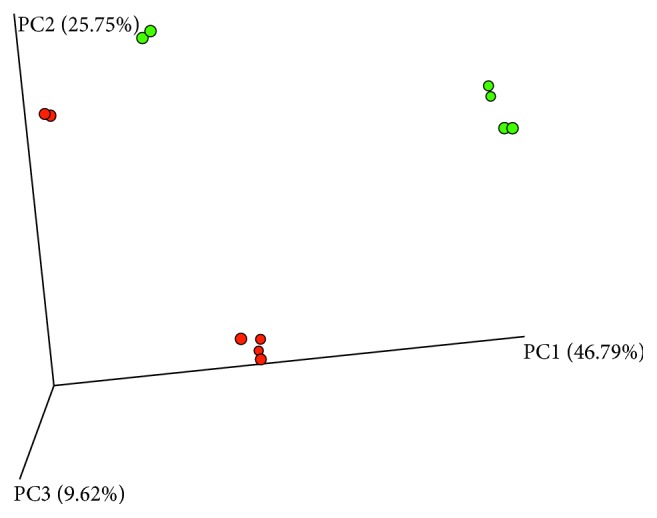
Principal coordinate analysis (PCoA, Bray–Curtis) plots of each sample as a point in a multidimensional space based on the composition of the bacterial population in each sample. Closeness of two points indicates similar bacterial population composition between the samples. PCoA plot of the fecal microbiome of three separate healthy episodes (green dots) and three HAEC episodes (red dots) with measurements performed in duplicate is given. Adonis and ANOSIM tests revealed statistically significant differences in the composition of the intestinal microbiome between healthy and HAEC episodes (Adonis: *p*=0.009, *R*^2^ = 0.31; ANOSIM: *p*=0.007, *R*^2^ = 0.52).

To assess beta diversity, a community analysis was performed by using principal coordinate analysis (PCoA) plots and Adonis and ANOSIM tests. A statistically significant difference in the composition of the fecal microbiome between healthy and HAEC episodes was found (Figure 1).

**Figure 2:**
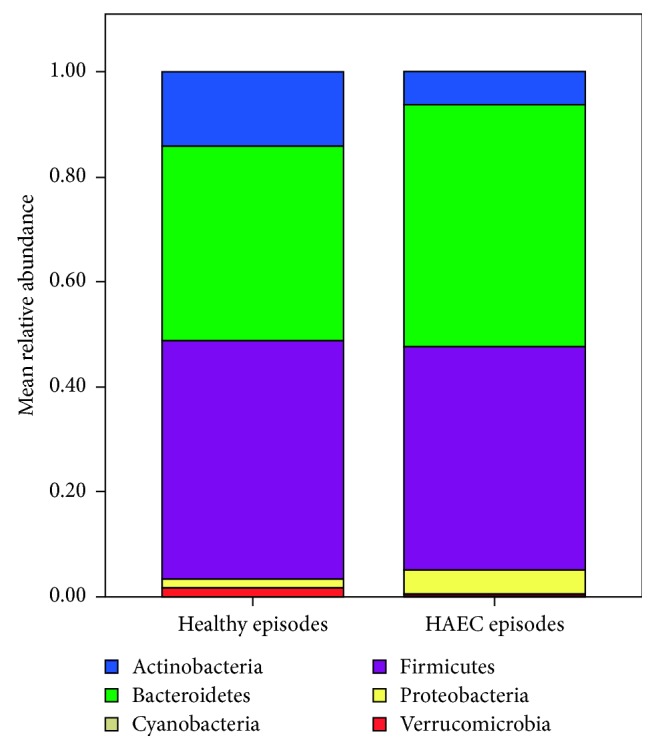
Mean relative abundances on the phylum levels comparing three healthy episodes and three HAEC episodes. Statistically significant changes were found for the phyla Actinobacteria, Bacteroidetes, Proteobacteria, and Cyanobacteria.

Taxonomic analysis revealed a statistically significant decrease of the relative abundances of Actinobacteria and significant increases of Bacteroidetes, Proteobacteria, and Cyanobacteria on the phylum level during HAEC episodes (Figure 2). A detailed overview of the statistically significant differences of the relative abundances on the remaining levels comparing healthy and HAEC episodes is given in Table 1. One of the most striking changes was seen for the genus *Bifidobacterium* which was reduced from 13% to 5% during HAEC episodes.

### 2.2. Probiotic Treatment

The patient was started on continuous treatment with probiotics for three months. In detail, he received one sachet of OMNi-BiOTiC® PANDA (Institut Allergosan, Graz, Austria) in the morning and one sachet of OMNI-BiOTiC® 10 AAD (Institut Allergosan, Graz, Austria) in the evening. OMNi-BiOtiC® PANDA contains *Lactococcus lactis* W58, *Bifidobacterium bifidum* W23, and *Bifidobacterium lactis* W52 (total of 3 × 10^9^ CFU/sachet). OMNi-BiOTiC® 10 AAD contains *Lactobacillus acidophilus W55*, *Lactobacillus acidophilus W37*, *Lactobacillus paracasei* W72, *Lactobacillus rhamnosus* W71, *Lactobacillus salivarius* W24, *Lactobacillus plantarum* W62, *Bifidobacterium bifidum* W23, *Bifidobacterium lactis* W18, *Bifidobacterium longum* W51, and *Enterococcus faecium* W54 (total of 5 × 10^9^ CFU/sachet). During these 3 months of treatment, fecal samples were taken weekly (as described above) adding to a total number of 14 samples.

During the observation period, the patient had episodes of diarrhea on 18% of the days (7 out of 39 days) without probiotic treatment and on 14% of the days under probiotic treatment (13 out of 90 days).

In the period of probiotic treatment, six stool samples were taken for microbiome analysis during diarrhea episodes and eight during healthy episodes. Probiotic treatment led to a significantly increased alpha diversity (Chao 1 Index) irrespective of healthy or HAEC episodes (mean healthy episode with probiotics 1,269, SD 111; mean HAEC episode with probiotics 1,274, SD 91; mean healthy episode without probiotics 967, SD 94; mean HAEC episode without probiotics 1,009, SD 72; *p* < 0.05 vs. their corresponding episode without probiotics).

**Figure 3:**
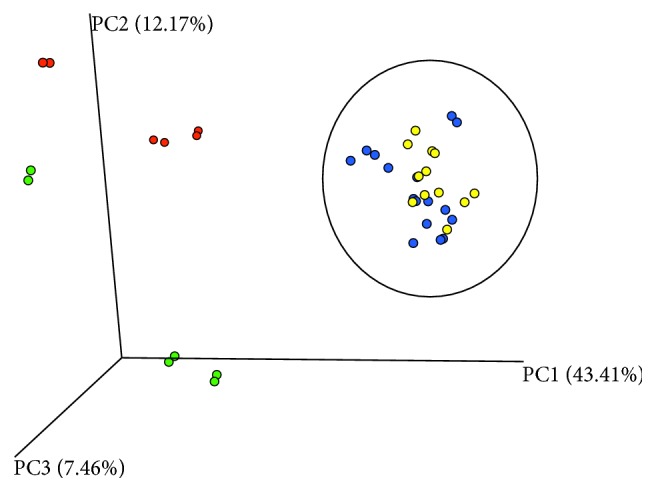
Principal coordinate analysis (PCoA, Bray–Curtis) of the fecal microbiome of healthy episodes (green dots) and HAEC episodes (red dots) before probiotic treatment and under probiotic treatment (healthy: blue dots, HAEC: yellow dots). Adonis and ANOSIM tests revealed statistically significant differences in the composition of the fecal microbiome between these episodes (Adonis: *p*=0.001, *R*^2^ = 0.49; ANOSIM: *p*=0.001, *R*^2^ = 0.98). Measurements were performed in duplicates.

Community analysis of the samples taken before and under probiotics is depicted in Figure 3. Statistically significant differences of the composition of the fecal microbiome were found between healthy and HAEC episodes and under probiotic treatment.

**Figure 4:**
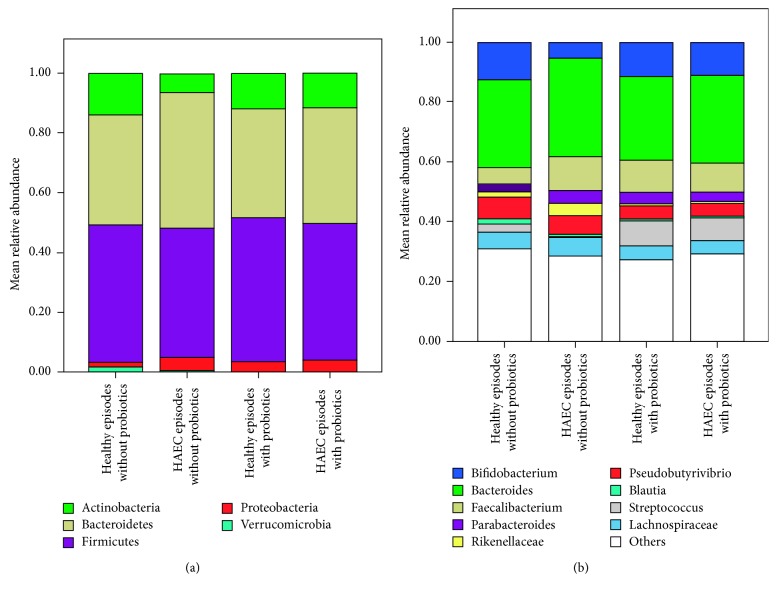
Mean relative abundances on the phylum levels (a) and genus level (b) before and under probiotic treatment in healthy and HAEC episodes. Note that on the genus level, only genera with more than 5% relative abundance are depicted.

Mean relative abundances on the phylum and genus levels are depicted in Figure 4. On the phylum level, the most striking findings were that, during HAEC episodes under probiotic treatment, the significant increase of Bacteroidetes and the decrease of Actinobacteria were not encountered (compare Figure 4). On the genus level, *Bifidobacterium* and *Streptococcus* were significantly increased during probiotic treatment. Additionally, probiotic treatment led to significant decreases of *Rikenellaceae*, *Pseudobutyrivibrio*, *Blautia*, and *Lachnospiraceae*.

## 3. Discussion

In a corrected HD patient, HAEC led to significant alterations of the fecal microbiome when compared to healthy episodes. Additionally, we were able to show that treatment with probiotics significantly alters the intestinal microbiome with the most striking changes observed comparing probiotic therapy to HAEC episodes. Moreover, these changes were not limited to the strains contained in the administered probiotics.

Even though a variety of different hypotheses have been formulated, the exact pathophysiology of HAEC still remains unclear. Recent studies have shown that a disruption of the intestinal mucosal barrier (“leaky gut”), an increase of inflammatory parameters, an abnormal immune response of the intestinal tract, and infection due to specific pathogens like *Clostridium difficile* may play pivotal roles in the development of HAEC [9]. However, both experimental and clinical studies recently have given a first insight into an altered intestinal microbiome in Hirschsprung's disease and HAEC and have revealed conflicting findings. For instance, Yan and coworkers have assessed the microbial signature of intestinal contents taken during surgery from different sections along the intestinal tract in a study population consisting of four patients (two patients with HAEC and two patients with Hirschsprung's disease) [5]. Bacteroidetes and Proteobacteria accounted for the highest proportion among the intestinal flora in Hirschsprung's disease patients. In contrary, Proteobacteria and Firmicutes were the most common microbes in HAEC patients. Comparing these results to those of the present study, we were able to find significant increases of Bacteroidetes and Proteobacteria during HAEC episodes. Nevertheless, changes of Firmicutes were not encountered in our patient (compare Figure 2). In another multicentric study consisting of patients suffering from Hirschsprung's disease, 9 with a history of HAEC and 9 without, the bacterial composition of the HAEC group showed a modest reduction in Firmicutes and Verrucomicrobia with increased Bacteroidetes and Proteobacteria compared to the group without HAEC [3]. Although these changes did not reach statistical significance, they are similar to the alterations found in our patient. However, all of the abovementioned studies were performed as interindividual comparisons, and therefore, biases influencing the intestinal microbiome caused by differences concerning geography, nutrition, and age cannot be ruled out. The present study reveals significant alterations of the fecal microbiome during HAEC episodes in an intraindividual comparison for the first time.

The fecal microbiome of the present patient during HAEC episodes indicates a proinflammatory state of the intestine. For instance, it has been proposed that an increased prevalence of Proteobacteria is a potential diagnostic signature of dysbiosis and risk of disease [10]. Other microbial diversity studies have also continually demonstrated an expansion of the Proteobacteria phylum in patients with inflammatory bowel disease [11]. It also has been shown that Actinobacteria can produce antibacterial agents [12]. Therefore, the decrease of their relative abundance during HAEC episodes most likely is associated with decreased levels of these agents fueling the proinflammatory intestinal state. However, it remains unknown whether these changes are a cause or a consequence of HAEC.

Treatment with probiotics was shown to be beneficial in a variety of diseases including acute infectious diarrhea, antibiotic-associated diarrhea, *Clostridium difficile*-associated diarrhea, hepatic encephalopathy, ulcerative colitis, irritable bowel syndrome, and necrotizing enterocolitis [13]. Regarding HAEC, however, the available reports are contradictory. While some studies have described that probiotics not only significantly diminish the incidence but also decrease the severity of HAEC [6], others did not confirm these findings [14]. Whether or not probiotic treatment can influence the occurrence of HAEC was not the focus of the present study, and answering this question would need prospective randomized trials. Nevertheless, analyses of the fecal microbiome of Hirschsprung's disease patients during probiotic treatment have not been performed yet. While the defecation pattern was not changed in our patient, we were able to describe alterations of the fecal microbiome during probiotic treatment with the most profound changes compared to HAEC episodes without probiotics. It is not surprising that the DNA of the applied probiotics can be detected in the feces of the treated patients. However, the fact that also other genera such as *Streptococcus*, *Rikenellaceae*, and *Blautia* were affected by probiotic treatment supports a broader influence of oral probiotic treatment on the gastrointestinal microbiome.

The main limitation of the present study remains the fact that it only presents data of one case. Nevertheless, we were able to compare within this patient three separate episodes of HAEC (APSA grade I) versus three healthy episodes. Additionally, a total of 14 investigations of the fecal microbiome during a three-month probiotic treatment period are included. Thus, this manuscript presents an innovative and important clinical concept inviting future studies including more patients. In conclusion, for the first time, this case presents the effective treatment of a child with HD and postoperative episodes of HAEC. Additionally, profound changes of the microbial composition during three months of probiotic treatment were found.

## Figures and Tables

**Table 1:** Relative bacterial abundances (%) on the different levels of the different episodes (*n*=3 healthy and *n*=3 HAEC). From the class level downstream, only germs with statistically significant differences (*p* < 0.05) are listed.

Phylum	Class	Order	Family	Genus	Healthy	HAEC	*p* value
Actinobacteria					14.09	6.25	0.004
	*Actinobacteria*	*Bifidobacteriales*	*Bifidobacteriaceae*	*Bifidobacterium*	12.94	5.50	0.004
Bacteroidetes					37.47	46.30	0.003
	*Bacteroidia*	*Bacteroidales*			37.50	46.30	0.003
			*Bacteroidaceae*	*Bacteroides*	29.90	33.70	0.02
			*Rikenellaceae*		1.70	4.20	0.02
Cyanobacteria					0.03	0.29	0.02
	*4C0d-2*				0.03	0.29	0.02
		*YS2*			0.03	0.30	0.02
Firmicutes					44.90	42.10	0.2
	*Bacilli*	*Lactobacillales*			2.84	0.24	0.02
	*Clostridia*	*Clostridiales*	*Lachnospiraceae*	*Dorea*	1.70	0.60	0.004
				*Lachnospira*	0.50	1.60	0.02
			*Ruminococcaceae*	*Oscillospira*	0.40	0.80	0.006
				*Ruminococcus*	2.40	1.40	0.01
			*Veillonellaceae*	*Veillonella*	0.03	0.14	0.04
Proteobacteria					1.60	4.52	0.01
	*Alphaproteobacteria*				0.48	1.71	0.01
		*RF32*			0.48	1.70	0.01
	*Deltaproteobacteria*	*Desulfovibrionales*	*Desulfovibrionaceae*	*Bilophila*	0.12	0.46	0.004
	*Gammaproteobacteria*	*Pasteurellales*	*Pasteurellaceae*	*Haemophilus*	0.18	0.50	0.025
Verrucomicrobia					1.79	0.48	0.14
